# Level-Ground and Stair Adaptation for Hip Exoskeletons Based on Continuous Locomotion Mode Perception

**DOI:** 10.34133/cbsystems.0248

**Published:** 2025-04-22

**Authors:** Zhaoyang Wang, Dongfang Xu, Shunyi Zhao, Zehuan Yu, Yan Huang, Lecheng Ruan, Zhihao Zhou, Qining Wang

**Affiliations:** ^1^ Department of Advanced Manufacturing and Robotics, College of Engineering, Peking University, Beijing 100871, China.; ^2^ School of Automation Science and Electrical Engineering, Beihang University, Beijing 100191, China.; ^3^ Department of Electronic and Computer Engineering, Hong Kong University of Science and Technology, Hong Kong 999077, China.; ^4^ School of Mechatronical Engineering, Beijing Institute of Technology, Beijing 100081, China.; ^5^ Beijing Engineering Research Center of Intelligent Rehabilitation Engineering, Beijing 100871, China.

## Abstract

Hip exoskeleton can provide assistance to users to augment movements in different scenarios. The assistive control for hip exoskeleton involves the interactions among exoskeleton, user, and environment, which depends on the environment perception (to predict locomotion) to design control strategy combined with gait mode and so on. Current exoskeleton control still needs to be improved in adaptation to continuous locomotion mode and different users. To address this problem, we have employed a learning-free (i.e., non-data-driven) environment perception method to improve hip exoskeleton adaptive control toward continuous locomotion mode. The adaptive control experiments were conducted on level ground and stairs on 7 subjects. The prediction accuracy for steady locomotion mode was more than 95% for each subject (ranged from 95.7% to 99.7%). The prediction accuracy for each locomotion mode transition ranged from 87.5% to 100%, and the transition timing could be detected before the end of transition period. Compared with learning-based (data-driven) approaches, our method achieves better performances in adaptive control for hip exoskeleton and shows some generalization for subjects.

## Introduction

Lower-limb exoskeleton has received much attention, as it is promising to enhance human locomotion capabilities and strength, reduce human’s effort, and so on [1–5]. Human’s lower-limb locomotion in daily activities involves various steady locomotion modes [level ground walking (LG), stair ascent (SA), stair descent (SD), and so on] and transitions from one locomotion mode to another locomotion mode. The lower-limb kinematics and kinetics show differences across locomotion modes and people [6–9].

Therefore, the importance of exoskeletons underscores the need for sophisticated control to realize the adaptive control for different locomotion and exoskeleton users.

The current exoskeleton adaptive control for different locomotion modes mainly adopts a finite state machine approach, which switches control strategy based on predicted locomotion mode [10–12]. The challenge of seamless control strategy transitions becomes increasingly apparent [13–17]. Traditional methods, such as manual mode switching [15], voice recognition and switching [18], or finger movement switching [19], rely on predefined control policies or users’ input, which limits their ability to respond to unforeseen environmental changes or user preferences [14], and lack the adaptation to various terrains.

To address the shortcomings of traditional control methods, researchers have embarked on a multifaceted exploration of alternative approaches to terrain perception and control adaptation. These avenues focus on the utilization of wearable sensors, such as inertial measurement units (IMUs) [11,20–22] and bio-electrical sensors [e.g., surface electromyography (sEMG)] [16], to sense terrain characteristics. These sensors provide a wealth of data for adjusting locomotion mode in response to the varying environmental conditions and have good prediction performance for continuous locomotion mode. For example, Huo et al*.* [14] have conducted the fast gait mode detection for an exoskeletal robotic orthosis and have good recognition performance. Despite the potential advantages offered by these research studies, some common limitations still need to be overcome. First, locomotion mode prediction relies on data-driven approach that might require training with specific datasets. For these methods to achieve high performance, datasets from the same unknown distribution with the environment are needed, which may affect its adaptability in unseen environments [23]. Second, while current locomotion mode prediction methods have shown good performance, there is still room for improvement, especially in predicting transitions between locomotion modes [24]. These factors can influence the adaptability of lower-limb exoskeleton control.

To overcome these limitations caused by data-driven approach and improve locomotion prediction performance for exoskeleton control, Qian et al*.* [24] have conducted terrain-adaptive exoskeleton control with gait mode recognition based on vision. For gait mode transition during LG and SA (no SD), their recognition accuracy is above 98.5% and recognition delay is at least 0.232 ± 0.040 gait cycle prior to the beginning of the transitional gait cycle [24]. Tricomi et al*.* [25] have conducted environment-based assistance modulation study for a hip exosuit via vision, while they only achieved more than 85% overall accuracy per locomotion mode.

To further these studies and improve the adaptive control performance of exoskeleton in LG, SA, and SD, we carried out the adaptive control study for hip exoskeleton based on a learning-free method for environment perception to improve the control adaptation to different terrains. In our previous study, we designed a novel learning-free locomotion mode prediction method for human locomotion without exoskeleton, which incorporated terrain reconstruction, and visual–inertial odometry (VIO) techniques, which could omit data generation and training process in machine learning-based algorithms and were robust to terrain and human [26]. In this study, a 3-level controller framework was designed to provide different functions for adaptive control of hip exoskeleton, including continuous mode prediction, gait phase detection based on the depth camera and other sensors integrated in exoskeleton, and control strategy design and execution. We conducted parameter pre-setting on the basis of public datasets [27] and continuous locomotion experiments on 7 subjects.

The contributions of this study as follows: (a) We design an exoskeleton control framework based on environment perception (level ground and stairs), which can effectively integrate environmental information and human or exoskeleton kinematic information, improve the detection accuracy and leading time of the transition, and contribute to improving switching smoothness of the exoskeleton control in different terrains. (b) We adopt a learning-free environment perception method for hip exoskeleton control. This exoskeleton adaptive control method can omit data collection for model training, which is not subjected to training data availability or parameter tuning for different people (i.e., non-data-driven and general for subjects), and maintain good prediction performance for continuous locomotion across terrains.

## Methods

### Hip exoskeleton design

**Fig. 1.**
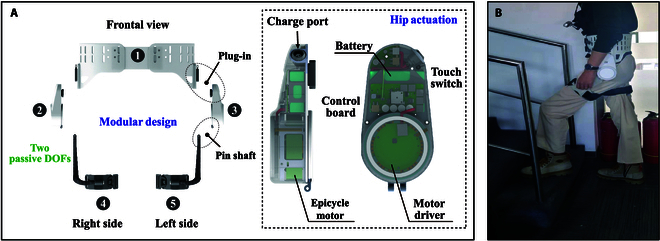
Exoskeleton design and wearing diagram. (A) Modular design and actuation of hip exoskeleton (including transmission, control and actuation components, etc.). (B) Wearing diagram.

The modular design of hip exoskeleton can be seen in Fig. 1A, which was composed of a waist belt, 2 sides of joint motor units, and 2 sides of thigh cuffs. The joint actuator included a direct current brushless motor and driver, control unit, and battery. The control unit was composed of a control circuit to collect signals and make decision to guide the exoskeleton action. The sensors in hip exoskeleton were composed of one depth camera (realsense D455, including RGBD camera and built-in IMU), pressure insole, angle sensor, and others. The camera was put on the user’s chest, and the hip exoskeleton was fixed on the subject’s waist through a strap device, as shown in Fig. 1B. Finally, the torque was transmitted to make the hip flexion and extension. The whole system was integrated on PC through ROS (Robot Operation System, noetic version).

### Multi-level controller design

**Fig. 2.**
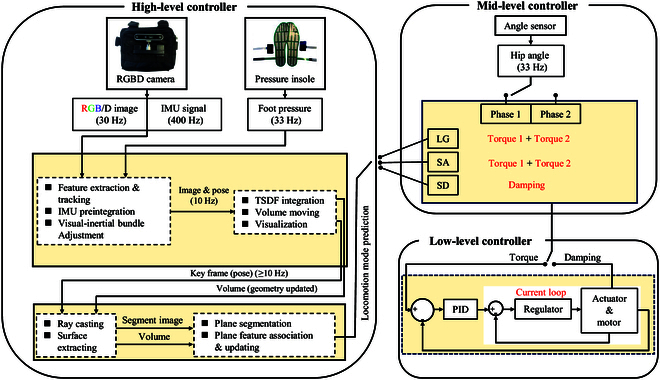
A 3-level controller designed for hip exoskeleton control. The high-level controller is designed to perceive environment and predict continuous locomotion mode, etc. The mid-level controller is designed to build the map from locomotion to control, namely, making control strategy based on the locomotion mode and gait phase. The low-level controller is designed to drive the motor and actuator to produce control torque or damping according to the set control strategies to provide assistance to human.

A 3-level controller framework was adopted to conduct exoskeleton control study, which could be seen in Fig. 2. It was composed of high-, mid-, and low-level controllers. Different controllers were designed for different purposes and functions, which reflected the interactions among environment, user, and exoskeleton.

#### High-level controller

High-level controller was designed to realize environment perception and predict continuous locomotion, as shown in Fig. 2. For environment perception, a depth camera was used to record the environment information including color information (red, green, blue), geometric information (depth), and inertial information (acceleration and gyroscope information) simultaneously.

We first adopted the depth-enhanced visual–inertial odometry system (D-VIO) algorithm to process the visual–inertial information obtained from camera to estimate the real-time pose (position and orientation) of the exoskeleton, which served as one critical foundation for subsequent mid- and low-level controller. This estimation was achieved through the fusion of visual and inertial data, facilitated by key frame-based optimization techniques and feature tracking mechanisms. By this approach, spatial relationship and interaction between exoskeleton’s movement and the surroundings could be updated continuously. Ultimately, the accurate estimation of the exoskeleton’s pose could be achieved.

The locomotion mode prediction was realized by a depth-enhanced VIO and a 3-dimensional (3D) reconstruction method referring to [26]. The goal of the D-VIO was estimating camera’s position, pose, and velocity. The D-VIO is formulated by

$$\underset{X_{W}}{\text{min}}C_{P}+C_{B}+C_{V}^{\mathrm{sw}}+C_{D}^{\mathrm{sw}}+C_{D_{h}}$$ (1)

where $X_{W}$ is the motion state consisting of camera position p, camera pose q, and camera velocity v; $C_{P}$ denotes the prior cost; $C_{B}$ denotes the IMU pre-integration cost; $C_{V}^{\mathrm{sw}}$ denotes the visual projection cost; $C_{D}^{\mathrm{sw}}$ represents depth measurement projection cost; and $C_{D_{h}}$ denotes the host depth cost. The details of each cost could be reviewed in [26]. With $X_{W}$, we can then merge depth images $I^{D}$ into a 3D environment map M. This method allows fast and accurate reconstruction of surfaces, from which we can extract planes with the PCA (principal components analysis) algorithm. As a result, we consider surfaces with a slope of less than $t_{\mathrm{lg}}$ as level ground, while adjacent surfaces with height differences are regarded as stairs.

The walking information, such as stride length, direction of movement, user footprint, and so on, can be estimated by kinematic states X. Pressure insoles play a crucial role in distinguishing footsteps by sensing pressure changes, which enables the collection of positional and velocity data. This process results in the accumulation of time series data for division position ${\left\{{\bar{p}}_{i}^{t}\right\}}_{i=1}$ and their related velocities ${\left\{{\bar{v}}_{i}^{t}\right\}}_{i=1}$, which can indicate the direction of movement as well. The measurement of step length δ between adjacent division position is computed using

$$\delta =∥\;{\bar{p}}_{i}^{t}-{\bar{p}}_{i-1}^{t}\;∥$$ (2)

where ${\bar{p}}_{i-1}^{t}$ and ${\bar{p}}_{i}^{t}$ refer to 2 adjacent positions in ${\left\{{\bar{p}}_{i}^{t}\right\}}_{i=1}$.

Using the last 2 step sizes, $\delta_{\text{step}}$ and $\delta_{\text{step}-1}$, the upcoming step size, known as $\delta_{\text{next}}$, can be determined through a first-order calculation method. This approach utilizes the previous measurements to provide an estimate for the next step’s size. Then, we can predict footprint position ${\bar{p}}^{\;fp}$ with $\delta_{\text{step}}$, $\delta_{\text{step}-1}$, and ${\bar{v}}_{i}^{t}$ as

$${\bar{p}}^{\;fp}={\bar{p}}_{i}^{t}+{\bar{v}}_{i}^{\mathrm{nm}}\cdot \left(2\delta_{\text{step}}-\delta_{\text{step}-1}\right)$$ (3)

where the unit vector ${\bar{v}}_{i}^{\mathrm{nm}}=\frac{{\bar{v}}_{i}^{t}}{|{\bar{v}}_{i}^{t}|}$ guarantees the scale invariance of $\delta_{\text{next}}$.

The distinction between the left and right feet is achieved by applying specific offsets to $p^{\;fp}$ for each foot. The locomotion mode can be predicted directly by comparing the height and slope between the current and predicted foot placements.

**Fig. 3.**
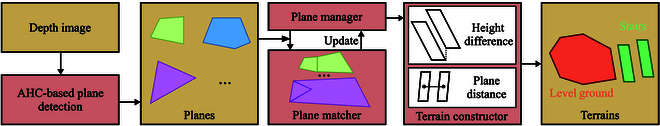
Terrain reconstruction process. Planes are extracted from depth images and matched with historical planes for plane management. Subsequently, terrains are reconstructed by comparing the distances and height differences between planes.

Terrain detection relied on an innovative algorithm rooted in normal vector and plane clustering methodologies, as shown in Fig. 3. In contrast to conventional machine learning approaches, our previous study capitalized on the intrinsic characteristics of environmental surfaces [26]. Upon receiving visual data from the integrated cameras, an in-depth analysis was conducted to discern the orientation of surfaces through normal vector analysis. This technique facilitated the differentiation of various terrains, such as level ground and stairs. Subsequently, plane clustering techniques were employed to group similar geometric attributes within the visual data. This clustering aided in the identification and classification of distinct terrain types. The resulting output of this thread provided a precise categorization of the prevailing terrain type. This information played a pivotal role in shaping the subsequent adjustment of exoskeleton control strategies.

Within the exoskeleton control paradigm, an indispensable element involved the detection of user’s gait phase. This critical function was executed through the utilization of a gait analysis algorithm that processed pressure data acquired from insoles. The algorithm processed the pressure data to extrapolate the user’s intended locomotion modes. By detecting dynamic variations of foot pressure distribution, the algorithm was capable of predicting the user’s intended movements, spanning from walking to ascending and to descending.

#### Mid-level controller

The mid-level controller was designed to link the high-level controller and low-level controller, which could also be viewed as a transition layer, and its function was to tailor proper control strategy according to the predicted locomotion mode and then design the control curve.

**Fig. 4.**
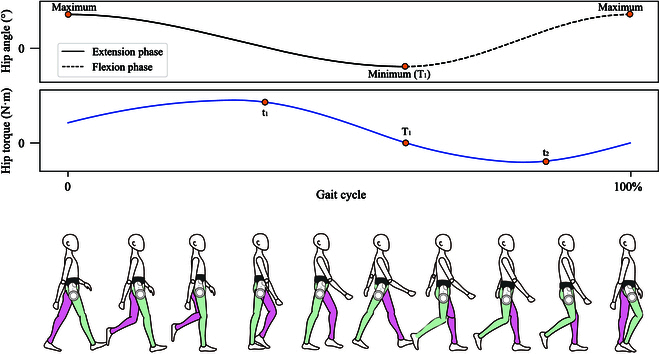
Hip angle, torque, and human locomotion during different gait phases in one gait cycle for hip exoskeleton. The black curve denotes hip angle, and the orange dots denote the maximum and minimum. The blue curve denotes hip torque, and the orange dots denote the critical torque values.

For each specific locomotion mode, control strategy varied in different gait phases as the corresponding biomechanical characteristics were different in each gait phase. For hip exoskeleton, we divided each gait cycle into 2 phases (extension phase and flexion phase), as shown in Fig. 4. The extension phase started at the maximum hip angle timing and ended at the minimum hip angle timing in each gait cycle. The flexion phase started at the minimum hip angle timing and ended at the maximum hip angle timing in each gait cycle.

Two main control strategies were considered according to people’s walking biomechanical characteristics. Based on public datasets [27], we analyzed the common characteristics of users with different physical signs. The control strategies of LG, SA, and SD were designed based on all or a part of these variables: maximum hip angle timing (denoted as $T_{1}$), extension peak phase timing (denoted as $t_{1}$), and flexion peak phase timing (denoted as $t_{2}$), as shown in Fig. 4 and Table 1. For LG and SA, different torque control strategies were adopted in extension and flexion phases to provide support and ensure safety, which could be seen as follows.

$$\begin{array}{c} \tau \left(x\right)=\left\{\begin{array}{c} A\text{sin}\left(\frac{\pi }{2t_{1}}x+\phi_{1}\right)\;0\leq x<t_{1}\\ A\text{sin}\left(\frac{\text{π}}{T_{1}}x+\phi_{2}\right)\;t_{1}\leq x<T\\ B\text{sin}\left(\frac{\pi }{2t_{2}}x+\phi_{3}\right)\;T_{1}\leq x<t_{2}\\ B\text{sin}\left(\pi x+\phi_{4}\right)\;t_{2}\leq x<100\% \end{array}\right. \end{array}$$ (4)

where A and B are the extension peak torque and flexion peak torque, respetively; x is the gait timing; $T_{1}$, $t_{1}$, and $t_{2}$ are some critical gait or torque timing as shown in Fig. 4 and Table 1; and $\phi_{1}$, $\phi_{2}$, $\phi_{3}$, and $\phi_{4}$ are the phase bias. For SD, damping control was employed during the whole gait cycle to provide support for hip joints to make the user go downstairs in a stable and safe way.

**Table 1.** Definition of gait timing and phase for exoskeleton control

Variables	Definition
$T_{1}$	Minimum hip angle timing
$t_{1}$	Extension peak torque timing
$t_{2}$	Flexion peak torque timing
Extension phase	From the maximum hip angle timing to the minimum hip angle timing
Flexion phase	From the minimum hip angle timing to the maximum hip angle timing

#### Low-level controller

The low-level controller was designed to drive motor and actuator to generate torque and damping, and then provide assistance to hip exoskeleton according to the set control strategy. For tracking the torque according to the given desired torque curve in the gait cycle, the torque was generated by the controller, which was composed of a torque loop with an inner current loop.

##### Torque control

According to the desired torque and the current assistive torque emanating from the actuators themselves, the torque difference was calculated and then output to the torque controller. This torque controller embodied the proportional-integral-derivative (PID) control framework. By comparing the desired current with the feedback motor current, the current difference was calculated and flowed to the current regulation, and finally motor and actuator could generate assistive torque.

##### Damping control

The damping control was realized by the shorted motor winding modulated by the pulse width modulation (PWM) signal to generate braking torque. By setting the duty cycle (D), different damping performance could be achieved according to the following formulation.

$$\tau_{d}={\mathrm{Dk}}_{d}\omega$$ (5)

where $k_{d}$ is the proportionality coefficient and ω is the rotation speed of the motor. The duty cycle (D) was customized and directly delivered to motor and the actuator to generate assistance/resistance.

In summary, this 3-level controller framework integrated VIO-based perception, terrain detection, and locomotion prediction to generate optimized control strategies for the exoskeleton, which empowered the exoskeleton system to effective interaction with environment, thereby leading to safe and smooth exoskeleton-assisted locomotion across different terrains.

### Experiment protocols

#### Pre-experiment for parameter setting

The torque control of hip exoskeleton depended on some parameters. To achieve these parameters, pre-experiments for parameter setting were conducted on a public dataset [27]. This dataset collected the kinematics and kinetics information (such as joint angle, torque, and so on) of more than 20 healthy subjects’ free walking on different terrains (level ground, stairs, and so on). For LG and SA, we calculated extension phase, flexion phase, extension peak torque timing, and flexion peak torque timing. Based on the hip angle and torque data from a subset of subjects, we estimated the normalized complete gait cycle, defined as the period from one heel strike to the next. Within this cycle, the proportion of the extension phase ($P_{\text{ext}}$) was calculated as the ratio of the average time from heel strike to toe-off (i.e., the time during which the hip extends from the maximum angle to the minimum angle) to the total gait cycle duration. The proportion of the flexion phase ($P_{\text{flex}}$) was determined as $100\%-P_{\text{ext}}$. Furthermore, we analyzed the timing of peak moments during these phases. The average time at which the peak moment occurs in the extension phase ($t_{1}$) and in the flexion phase ($t_{2}$) was expressed as percentages of the respective phase durations. The calculated results are listed in Table 2.

**Table 2.** Assistive strategies parameters

Locomotion mode	Control strategy	Extension phase	Flexion phase	Extension peak torque timing ($t_{1}$)	Flexion peak torque timing ($t_{2}$)
LG	Torque	0–53%	53–100%	42%	84%
SA	Torque	0–62%	62–100%	37%	87%
SD^^a^^	Damping	-	-	-	-

^a^ Damping control is designed in the SD across the whole gait cycle, which does not rely on these gait parameters in this table, so the value of parameters is not listed.

After the above calculation process, we concluded that (a) for LG, the extension phase ranged from 0 to 53% of the gait cycle, and the flexion phase ranged from 53% to 100% of the gait cycle, as listed in Table 2. The peak torque output during the extension appeared at about 42% of the gait cycle, and the peak torque output in the flexion phase appeared at about 84% of the gait cycle. (b) For SA, the extension phase ranged from 0 to 62%, and the flexion phase ranged from 62% to 100% in the gait cycle. The peak torque timing during the extension phase occurred at nearly 37% of the gait cycle, and the peak torque timing during the flexion phase occurred at nearly 87% of the gait cycle. (c) For SD, we designed the constant damping mode during the whole gait cycle to make the user go downstairs in a stable and safe way, which did not rely on these gait parameters.

#### Hip exoskeleton continuous locomotion experiments

The objectives of experiments were to predict locomotion mode and transition first and then conduct hip exoskeleton control based on the predicted locomotion. We recruited 7 healthy subjects (6 males and 1 female) for the continuous experiments. Their average height was 177.4 ± 8.6 cm, and their average weight was 75.4 ± 7.6 kg (more details can be seen in Table 3). All these subjects provided informed written consents, and the experiments have been approved by the Local Ethics Committee of Peking University.

**Table 3.** Information of 7 healthy subjects

ID	Gender	Age	Weight/kg	Height/cm
S01	Male	23	75	188
S02	Male	26	75	181
S03	Male	32	80	177
S04	Male	21	85	179
S05	Male	22	80	180
S06	Male	21	74	179
S07	Female	22	59	158

In experiments, each subject wore the integrated hip exoskeleton, and the depth camera was put on each subject’s chest to perceive environment, and the hip exoskeleton was fixed on the subject’s waist through a strap device. The outlines of these experiments were that each subject walked on level ground and stairs to finish 3 steady locomotion modes (LG, SA, and SD) and 4 transitions (LG→SA, LG→SD, SA→LG, and SD→LG). The experiment for each subject contained 20 trials. In each trial, subject wore the exoskeleton and walked on level ground (LG: 2 gait cycles), transition to stairs (LG→SA: 1 gait cycle), walked on stairs (SA: 2 gait cycles), transition to level ground (SA→LG: 1 gait cycle), and walked on level ground (LG: 2 gait cycles). Then, the subject turned around to finish the continuous locomotion from on level ground to stairs to level ground (LG: 2 gait cycles, LG→SD: 1 gait cycle, SD: 2 gait cycles, SD→LG: 1 gait cycle, and LG: 2 gait cycles). During experiments, visual and inertial signals from the depth camera, foot pressure, hip angle signals, and some other signals were collected. All the signals were packed and then transmitted to the PC. All the data were processed, and locomotion mode prediction was conducted based on the sensor data.

### Performance evazluation

The performance evaluation of level-ground and stair adaptation for hip exoskeletons mainly included continuous locomotion mode prediction on different terrains, which could be quantified using prediction accuracy metric and transition timing. Continuous locomotion mode was consisted of steady locomotion mode and mode transition from level ground/stair to stair/level ground. The prediction accuracy for steady locomotion mode was calculated according to the percentage of the correct predicted numbers in all sample numbers. Higher accuracy indicated better capacity to accurately predict locomotion modes.

For locomotion mode transition, the transition period started at the heel strike and ended at the next heel strike, which lasted for one gait cycle. Here, we used ($T_{\text{start}}$) and ($T_{\text{end}}$) to represent the start timing and end timing of transition period. One correct transition prediction was described as follows. For transition from mode A to mode B, if the upcoming locomotion mode B was predicted at one timing ($t_{k}$) before the end timing of transition period and no other locomotion modes were predicted from $t_{k}$ to the end timing of transition, the transition from mode A to mode B was predicted correctly.

The prediction accuracy for transition was the percentage of correctly predicted transition numbers in all transition numbers. Moreover, it could be seen that the correctly predicted transition timing was before the end timing of transition. Therefore, one predicted time metric was designed to describe the time deviation from the predicted transition timing to the end timing of transition, which was formulated as follows.

$$\Delta_{t}=t_{k}-T_{\text{end}}$$ (6)

It could be seen that $\Delta_{t}\leq 0$. We could use the absolute value of $\Delta_{t}$ (i.e., $|\Delta_{t}|$) as the predicted leading time. The ratio of predicted leading time ($|\Delta_{t}|$) to transition period ($T_{\text{end}}-T_{\text{start}}$) was the predicted leading time ratio (denoted as $T_{\text{ratio}}$), which was calculated as follows.

$$T_{\text{ratio}}=\frac{|\Delta_{t}|}{T_{\text{end}}-T_{\text{start}}}$$ (7)

$T_{\text{ratio}}$ ranged from 0 to 100%. Higher accuracy and larger time ratio indicated better capacity to accurately predict transitions from level ground/stair to stair/level ground.

## Results

### Locomotion signals on level ground and stairs

**Fig. 5.**
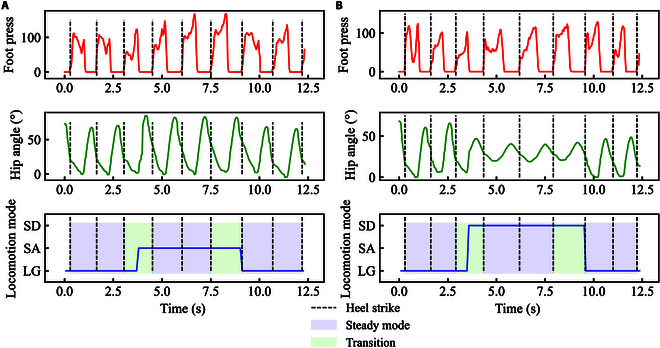
The locomotion signals (foot press, hip angle, and locomotion mode) on level ground and stairs. (A) Foot press and hip angle during continuous locomotion mode (LG, LG→SA, SA, and SA→LG). (B) Foot press and hip angle during continuous locomotion mode (LG, LG→SD, SD, and SD→LG). The red, green, and blue curves denote the foot press, hip angle, and predicted locomotion mode and transition. The black dashed lines denote the heel strike in each gait cycle detected by foot press. The light blue and green regions in the third row of (A) and (B) denote the steady locomotion mode and transition period, which can be viewed as the ground truth of locomotion mode and transition. Here, we present foot press and hip angle of right lower limb during continuous locomotion. Data come from the first subject (S01).

The locomotion signals, including foot press, hip angle, and locomotion mode during continuous locomotion, could be seen in Fig. 5. Based on the foot press signals, we could get the heel strike event and then divide the gait cycle. Besides, taking these gait cycles and terrain geometry setting into consideration, the ground truth of locomotion mode and transition period was calculated. In each gait cycle, the hip angle exhibited differently in continuous locomotion mode.

From level ground to stair and to level ground (upstairs), there were 2 steady locomotion modes (LG and SA) and 2 transitions (LG→SA and SA→LG), as shown in Fig. 5A. The corresponding peaks of hip angle during SA were larger than those during LG in gait cycles. Besides, we could see that there existed obvious transition during transition period. The transition (LG→SA) timing was before the end timing of transition period, and the transition (SA→LG) timing was close to the end timing of transition period (see Fig. 5A). From level ground to stair and to level ground (downstairs), there were 2 steady locomotion modes (LG and SD) and 2 transitions (LG→SD and SD→LG), as shown in Fig. 5B. The corresponding peaks of hip angle during SD were smaller than those during LG in gait cycles. The transition (LG→SD) timing was before the end timing of transition period, and the transition (SD→LG) timing was close to the end timing of transition period, as shown in Fig. 5B).

### Prediction accuracy for steady locomotion mode

**Fig. 6.**
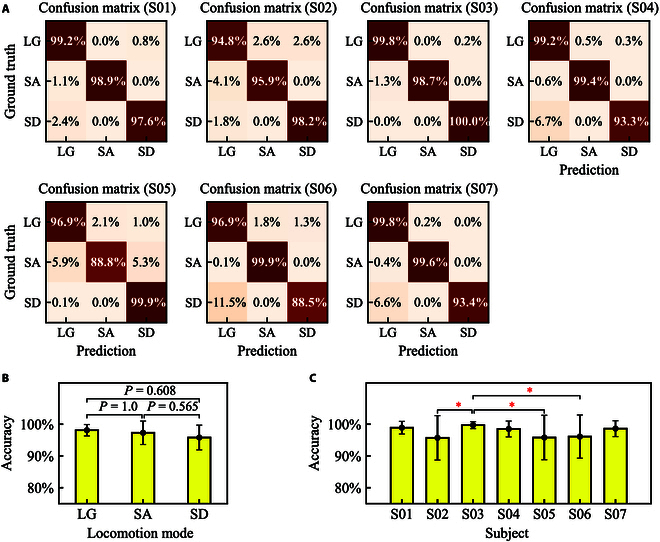
The steady locomotion mode prediction performance for hip exoskeleton. (A) Confusion matrix of steady locomotion mode for each subject. (B) Prediction accuracy (mean ± SD) for each locomotion mode. The *P* values are annotated based on Mann–Whitney *U* test. (C) Prediction accuracy (mean ± SD) for each subject. ${}^{*}$*P* < 0.05 (Welch’s *t* test). The error bars in (B) and (C) represent ±SD.

Confusion matrix was adopted to demonstrate steady locomotion mode prediction, as shown in Fig. 6A. For subject 1, the prediction accuracy for each locomotion mode was more than 95%, and the accuracy for LG, SA, and SD was 99.2%, 98.9%, and 97.6%, respectively. For subject 2, the prediction accuracy for LG, SA, and SD was 94.8%, 95.9%, and 98.2%, respectively. For subject 3, the prediction accuracy for LG and SA was 99.8% and 98.7%, respectively. Moreover, the prediction accuracy for SD was 100%. For subject 4, the prediction accuracy for LG, SA, and SD was 99.2%, 99.4%, and 93.3%, respectively. For subjects 5 and 6, there existed conditions that the prediction accuracy was lower than 90%, which was 88.8% (SA) for subject 5 and 88.5% (SD) for subject 6. For subject 7, the prediction accuracy for each locomotion mode was more than 90%.

The accuracy across 7 subjects for LG, SA, and SD was 98.1% ± 1.8%, 97.3% ± 3.7%, and 95.8% ± 3.9%, respectively, as shown in Fig. 6B. For each steady locomotion mode, the average of prediction accuracy was more than 95%. Mann–Whitney *U* test was adopted for performance difference analysis. There were no significant differences between LG and SA, SA and SD, and LG and SD (*P* values were 1.0, 0.565, and 0.608, respectively). The accuracy across 20 trials for each subject was 98.9% ± 2.0%, 95.7% ± 7.0%, 99.7% ± 1.1%, 98.5% ± 2.5%, 95.8% ± 7.0%, 96.1% ± 6.8%, and 98.6% ± 2.5%, respectively, as shown in Fig. 6C. For each subject, the average of prediction accuracy was more than 95%. For subject 3, the average of prediction accuracy was the largest and the standard deviation (SD) was the smallest. For subjects 2, 5, and 6, the average of prediction accuracy was smaller and the SD was larger. There were significant differences between subject 3 and subjects 2, 5, and 6 (Welch’s *t* test, *P* < 0.05).

### Prediction accuracy and leading time for transition

**Fig. 7.**
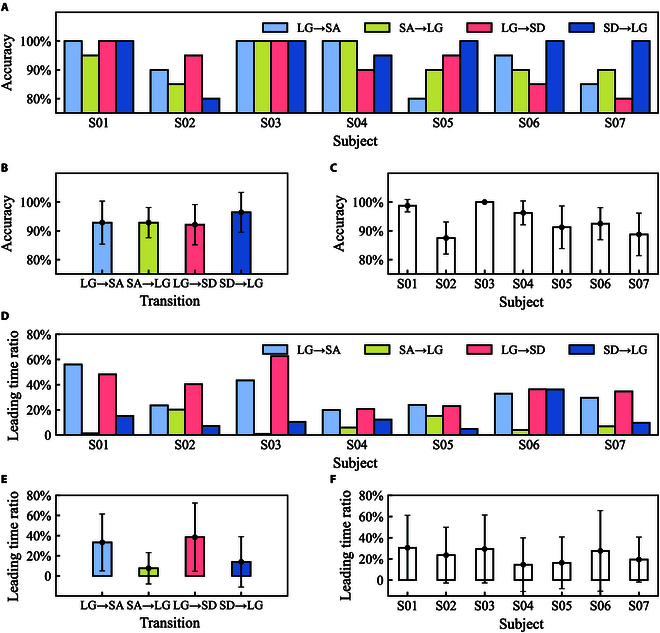
The transition prediction performance for hip exoskeleton. (A) Prediction accuracy for each transition and each subject. (B) Prediction accuracy (mean ± SD) for 4 types of transition across all subjects. (C) Prediction accuracy (mean ± SD) for each subject. (D) Predicted leading time ratio for each transition and each subject. (E) Predicted leading time ratio (mean ± SD) for 4 types of transition across all subjects. (F) Predicted leading time ratio (mean ± SD) for each subject. The error bars represent ±SD.

The prediction accuracy of locomotion mode transition served as one important metric, which could be seen in Fig. 7. For subject 1, the prediction accuracy for transition (SA→LG) was 95%, and we could get 100% accuracy for the other 3 transitions, as shown in Fig. 7A. For subject 2, the prediction accuracy ranged from 80% to 95% for transitions. For subject 3, each transition could be predicted with 100% accuracy. For subject 4, 2 transitions (LG→SA and SA→LG) could be predicted with 100% accuracy. For subjects 5, 6, and 7, one transition (SD→LG) could be predicted with 100% accuracy. The accuracy for 4 transitions was 92.9% ± 7.5%, 92.9% ± 5.2%, 92.1% ± 7.0%, and 96.4% ± 6.9%, respectively, as shown in Fig. 7B. For each subject, the mean accuracy was more than 85.0%. The accuracy for subject 3 was 100.0% ± 0.0%, which was the best, as shown in Fig. 7C. The mean accuracy for subject 2 was 87.5%, which was the smallest compared with others, as shown in Fig. 7C.

The predicted transition timing ratio could be seen in Fig. 7D. The maximum leading time ratio occurred in transition from LG to SD, which was 62.6% (subject 3). The minimum leading time ratio occurred in transition from SA to LG, which was 1.2% (subject 1). The leading time ratios for 4 transitions were 33.2% ± 28.2%, 7.5% ± 15.6%, 38.4% ± 34.0%, and 13.9% ± 25.0%, as shown in Fig. 7E. The mean leading time ratio for transition from SA to LG was the smallest compared with the other 3 transitions. The leading time ratios for all subjects were 30.5% ± 30.6%, 23.5% ± 26.3%, 29.4% ± 32.1%, 14.5% ± 25.3%, 16.3% ± 24.3%, 27.5% ± 38.1%, and 19.4% ± 21.2%, as shown in Fig. 7F. The maximum was 30.5% (subject 1), and the minimum was 14.5% (subject 4).

## Discussion

The frequently used exoskeleton control method depends on environment perception to predict locomotion mode and build and switch control strategy, which has some limitations to be overcome. For example, as the control strategies adopt data-driven locomotion prediction method, the prediction model and control strategies are customized for user individuals and lack of generality [11]. In addition, transition prediction performance is not enough and still needs to be improved in both accuracy and leading time. To address these problems, we conducted hip exoskeleton adaptive control study on level ground and stairs toward continuous locomotion based on a learning-free (i.e., non-data-driven) environment perception method in this study.

### Method and result analysis

This adaptive control study for hip exoskeleton is conducted based on a 3-level controller framework. The high-level controller is designed to realize environment perception. The mid-level controller is employed to make control strategy for each specific locomotion mode based on biomechanical characteristics. The control torque curve is generated in mid-level controller, and it is delivered to users in low-level controller. This study focuses more on improving high-level controller, which is designed to realize environment perception to predict continuous locomotion mode, omitting data dependent or parameter tuning for different subjects and maintaining good prediction performance across 7 subjects.

The prediction accuracy for each steady locomotion is more than 95%, and there are no significant differences between LG and SA, SA and SD, and LG and SD (Mann–Whitney *U* test, *P* > 0.05), as shown in Fig. 6B. The prediction accuracy for each subject is also more than 95%, as shown in Fig. 6C. Especially for subject 3, the prediction accuracy is up to 99.7% ± 1.1%, which exhibits significant difference between subject 3 and subjects 2, 5, and 6 (Welch’s *t* test, *P* > 0.05). There are no significant differences between other subjects.

The transition prediction accuracy ranges from 80% to 100% for each type of transition, and the average of accuracy was more than 87.5% (Fig. 7). The predicted transition timing occurred before the end of transition period. Its corresponding predicted leading time ratio ranges from 14.5% to 30.5% for different subjects. Compared to traditional locomotion mode recognition method [11,21,22,28,29], this could void some problems (gait instability and fall risk) caused by a delayed gait mode recognition in abrupt changes in assistive behaviors [30]. Both the transition prediction performances served as a critical metric for the control transition of exoskeleton between 2 different locomotion modes to modulate the exoskeleton assistance and ensure safety during transitions.

### Comparison with data-driven method

Tricomi et al*.* [25] have conducted assistance modulation study for a hip exosuit via computer vision based on convolutional neural networks (CNNs) on level ground and stairs, and the overall accuracy for each steady locomotion mode is more than 85% and the maximum transition prediction accuracy is 67.86% ± 8.99%. Compared with their study, our study has achieved better performances for both steady locomotion mode (from 95.7% to 99.7%) and transition prediction (from 87.5% to 100%). Qian et al*.* [24,31] have conducted locomotion mode recognition on various terrains and terrain-adaptive exoskeleton control study during level walking and SA (not including SD) based on CNN. Al-Dabbagh and Ronsse [32] have conducted depth vision-based terrain detection using machine learning during human locomotion (without exoskeleton), and recognition accuracy for the upcoming terrain is more than 95%. Compared with these vision-based data-driven methods, our method has also acquired comparable prediction performance. Different from these data-driven methods that require to collect data and train model for each subject, our learning-free method can omit data collection (i.e., non-data-driven) for model training and parameter tuning, which exhibits generalization for subjects.

### Limitation and future work

While this research provides insights in improving exoskeleton adaptive control, there are still some limitations to be addressed.

The limitations of adopted environment perception (vision-based) method mainly lie in sensing and algorithm decision. The vision-based method is subjected to lighting conditions, as changes in ambient light can impact terrain prediction. Besides, the system’s performance decreases when the visual input is obstructed, where the line of sight is blocked by obstacles. Additionally, stairs with a deep tread may be identified as a plane caused by the prediction algorithm, and the prediction still needs to be evaluated in complex or unstructured environment. All these can impact continuous locomotion prediction, which need further research to enhance the system’s robustness, potentially through sensing method and/or refining visual processing algorithm.

The limitations in experiments mainly lie in subject, terrain, and so on. More experiments need to be conducted on more subjects including healthy people and patients, and effects of exoskeleton adaptive control methods still need to be validated on other terrains, such as ramps.

In addition, some physiological metrics (such as sEMG and metabolic cost) need to be employed to evaluate the assistance improvement of our exoskeleton adaptive control method.

## Conclusion

In this study, we conducted a learning-free adaptive control study for hip exoskeleton without environment parameter setting for different users to improve the control adaptation to different locomotion modes. A 3-level controller framework was designed to realize environment perception, continuous locomotion mode prediction, gait phase detection in high level, control strategy generation in mid level, and torque execution in low level. The exoskeleton control experiments were conducted on level ground and stairs on 7 subjects. Prediction accuracy for steady locomotion mode was more than 95% accuracy for each subject. The locomotion mode transition could be predicted before the end of transition period with high accuracy (from 87.5% to 100%). Our study has provided an effective assistive control approach to improve the control adaptation of hip exoskeleton on level ground and stairs for different users.

## Data Availability

The data used for this study are available from the corresponding author upon reasonable request.
